# Heterozygous *MMACHC* burden variants are associated with higher circulating vitamin B12 in the *All of Us* Research Program

**DOI:** 10.64898/2026.06.03.26354855

**Published:** 2026-06-04

**Authors:** Ling Cai, Ralph J. DeBerardinis

## Abstract

Heterozygous carriers of autosomal recessive disease variants are conventionally considered unaffected, yet population-scale genomic datasets reveal subclinical carrier phenotypes. *MMACHC* encodes a cobalamin-processing protein whose biallelic loss causes cobalamin C deficiency, an inborn error of intracellular cobalamin metabolism. We performed an unbiased quantitative phenome-wide association screen in *All of Us* Research Program v8 to identify phenotypes associated with rare heterozygous *MMACHC* burden variants. Serum/plasma vitamin B12 was the top quantitative association. Carriers had higher circulating B12 than non-carriers in adjusted analyses, but also higher homocysteine, suggesting that elevated circulating B12 does not reflect improved intracellular cobalamin function. Carriers were less likely to fall below conventional B12 insufficiency thresholds, indicating a potential diagnostic blind spot. A pathway-wide rare-variant gene-burden (All-by-All) gene-burden analysis placed this finding in broader biological context. Burdens in genes related to circulating B12 binding or intestinal absorption were associated with lower circulating B12. In contrast, burdens in several genes involved in cellular delivery and intracellular cobalamin handling were associated with higher circulating B12. This step-specific directionality supports a model in which elevated circulating B12 can reflect impaired cellular handling and consequent systemic accumulation rather than improved cellular cobalamin availability. Because EHR-derived B12 is shaped by heterogeneous clinical and medication contexts, prospective carrier-enriched studies with standardized methylmalonic acid, homocysteine, diet, supplement, medication, comorbidity, and symptom ascertainment are needed to evaluate functional-marker-based screening.

## Full Text Availability

The license terms selected by the author(s) for this preprint version do not permit archiving in PMC. The full text is available from the preprint server.

